# 离子色谱-串联质谱法检测酒类产品中10种有机酸

**DOI:** 10.3724/SP.J.1123.2022.01020

**Published:** 2022-12-08

**Authors:** Yingqi MU, Yixuan WU, Xiao WANG, Liming HU, Runhui KE

**Affiliations:** 1.北京工业大学环境与生命学部, 北京 100124; 1. Faculty of Environment and Life, Beijing University of Technology, Beijing 100124, China; 2.中国食品发酵工业研究院有限公司, 北京 100015; 2. China National Research Institute of Food &Fermentation Industries Co., Ltd., Beijing 100015, China; 3.中轻检验认证有限公司, 北京 100016; 3. Sinolight Inspection &Certification Co., Ltd., Beijing 100016, China

**Keywords:** 离子色谱-串联质谱, 有机酸, 白酒, 黄酒, 干红葡萄酒, ion chromatography-tandem mass spectrometry (IC-MS/MS), organic acids, liquor, yellow rice wine, dry red wine

## Abstract

建立了一种离子色谱-串联质谱(IC-MS/MS)测定白酒、黄酒、干红葡萄酒3种酒类样品中10种有机酸含量的方法。白酒样品氮吹后,经去离子水稀释,用IC-MS/MS分析检测;干红葡萄酒样品和黄酒样品,对比不同固相萃取小柱净化能力,最终选择石墨化炭黑固相萃取小柱进行净化,经去离子水稀释,用IC-MS/MS分析检测。选用高容量、强亲水性的Dionex IonPac^TM^ AS11-HC型阴离子分析柱进行分离,以淋洗液自动发生器在线产生的KOH水溶液为淋洗液,进行梯度淋洗。淋洗液经抑制器抑制后直接进入电喷雾电离串联质谱(ESI-MS/MS),采用负离子模式电离,多反应监测(MRM)模式检测,外标法定量。在该实验条件下:草酸、富马酸、马来酸、苹果酸、酒石酸、柠檬酸、奎尼酸和乌头酸在0.05~2 mg/L范围内线性关系良好;琥珀酸在0.05~5 mg/L范围内线性关系良好;乳酸在0.05~10 mg/L范围内线性关系良好(相关系数*r*^2^>0.99)。10种有机酸的检出限(*S/N*=3)在1.0~8.0 μg/L范围内,定量限(*S/N*=10)在3.5~26.5 μg/L范围内,在3个不同浓度的添加水平下,平均回收率在83.0%~112.1%之间,相对标准偏差≤9.1%,满足检测要求。该方法前处理简单,不使用有机溶剂,不需进行衍生化处理,测定快速、准确,灵敏度高,适用于3种酒类样品中10种有机酸的定量分析,为酒类食品的风味及品质测定提供方法支持。

## Abstract (en)

A method was developed for the determination of 10 organic acids in liquor, yellow rice wine, and dry red wine by ion chromatography-triple quadrupole mass spectrometry (IC-MS/MS). First, the liquor samples were diluted with deionized water, degassed with nitrogen, and analyzed by IC-MS/MS. Then, the yellow rice wine and dry red wine samples were purified with different solid-phase extraction cartridges. Finally, the GCB solid-phase extraction cartridge was selected for purification, diluted with deionized water, and analyzed by IC-MS/MS. The samples were separated using a Dionex IonPac AS11-HC anion analysis column with high capacity and strong hydrophilicity, with an KOH aqueous solution as the eluent, which was produced by an automatic generator for gradient elution. After being suppressed using a suppressor, the eluent was injected directly into the electrospray ionization tandem mass spectrometry (ESI-MS/MS), ionized in negative ion mode, detected in multiple reaction monitoring (MRM) mode, and quantified using an external standard method. Oxalic acid, fumaric acid, maleic acid, malic acid, tartaric acid, citric acid, quinic acid, and aconitic acid showed good linear relationships in the range of 0.05-2 mg/L. Succinic acid and lactic acid showed good linearities in the range of 0.05-5 mg/L and 0.05-10 mg/L, respectively. The correlation coefficients (*r*^2^) were >0.99. The limits of detection (LODs) and limits of quantification (LOQs) were 1.0-8.0 μg/L and 3.5-26.5 μg/L, respectively. The average recoveries ranged from 83.0% to 112.1%, and the relative standard deviations (RSDs) were <9.1% in spiked samples at three levels. The proposed method allowed easy pretreatment without using organic solvents or derivatization processing. Overall, the proposed method is accurate, rapid, sensitive, and it is suitable for the qualitative and quantitative analyses of the 10 organic acids in three wine samples. Moreover, it can be used for the determination of flavor and quality of alcoholic products.

有机酸是一类含有羧基的酸性化合物,存在广泛,种类繁多,是重要的生理活性物质^[1]^。同时,有机酸还有助于增加食品的感官特性和健康特性,对食品的品质控制起到重要作用^[2]^。

有机酸是酒类口味的重要组成部分,赋予酒体特定的口感和味道,有机酸的缺乏或者过量都会导致酒的口感不协调^[3]^。不同的原料与酿造工艺使不同类型的酒中有机酸组成和含量有一定的差别,对酒的感官品质有很大影响,是酒类产品风味与营养的质量指标^[4]^。因此,有机酸的分析对酒类酿造过程的调控及成品的评定与质量监控都有重要意义。但是,对于复杂基质中低含量的目标物进行有效分离和准确测定是酒中有机酸检测时所面临的重大挑战^[5]^。

常用的有机酸检测方法有液相色谱法、液相色谱-串联质谱法、气相色谱法、毛细管电泳法、离子色谱法(IC)等^[6⇓⇓⇓-10]^。液相色谱法中部分有机酸会出现共洗脱现象^[11]^,分离效果较差;液相色谱法与液相色谱-串联质谱法在测小分子有机酸时均需要用极性大、pH低的洗脱液,对色谱柱破坏较大,不宜批量检测^[12]^;气相色谱法适用于相对分子质量较低挥发性有机酸的检测,对于相对分子质量较高的有机酸则需要进行衍生化处理,操作烦冗,重复性较差,质量控制相对较难^[13]^;毛细管电泳法检测速度快、实验成本低,适用于有机酸的快速分析,但相比于色谱法,其精密度和灵敏度较差^[14]^。相对来说,离子色谱法是分析有机酸的一种有效方法,具有操作简便、分离良好、灵敏度高等优点^[15]^,但其局限性在于目标物的定性只能依靠保留时间,且易受其他共存离子的干扰^[16,17]^。三重四极杆质谱凭借抗干扰能力强、灵敏度高、选择性好、适用范围广等优势,已成为痕量物质定性确证和定量分析的首选方法^[18]^。通过联用,离子色谱-串联质谱仪(IC-MS/MS)将离子色谱与三重四极杆质谱的优点相结合,既提高了极性物质的分离能力,又提高了在复杂基质中对极性物质检测的选择性与灵敏度,与此同时还能够提供物质的相对分子质量与结构信息。应用IC-MS/MS来分析酒中有机酸,不仅可以实现多种有机酸的同时测定,而且可以对其结构进行确证,提高检测的灵敏度和准确性。目前,采用固相萃取法对酒中有机酸进行样品前处理,并结合IC-MS/MS对有机酸进行分析的研究鲜有报道。

本研究通过设计新型的连接流路,解决了离子色谱与质谱相连接时所面临的流速不兼容及缓冲盐污染质谱的难题,搭建了离子色谱-三重四极杆质谱串联的分析装置,以酒类样品中有机酸为切入点,探索离子色谱-串联质谱技术在极性物质检测中应用的有效性和实用性。同时,对样品前处理技术、色谱分离条件、质谱检测参数等做了探讨研究,建立了一种高效、准确、绿色测定酒类样品中有机酸的方法,极大地降低了基体干扰,提高了分析方法的信噪比和灵敏度,为酒类品质管理和质量评价提供了强有力的方法支持。

## 1 实验部分

### 1.1 仪器、试剂与材料

Dionex ICS3000型离子色谱仪(美国Dionex公司); ACQUITY TQD三重四极杆质谱仪(美国Waters公司); Dionex IonPac^TM^ AS11-HC型阴离子分析柱(250 mm×4 mm)、Dionex IonPac^TM^ AS11-HC型保护柱(50 mm×4 mm)、Dionex ADRS 600 (4 mm)阴离子抑制器(美国Thermo公司); Milli-Q纯水仪(美国Millipore公司);涡旋混合器(德国IKA公司);氮吹仪(北京帅恩科技有限公司)。

草酸、富马酸、马来酸、琥珀酸、苹果酸、酒石酸、柠檬酸、奎尼酸、乌头酸和乳酸(纯度>98%,坛墨质检科技股份有限公司); IC-RP固相萃取柱和石墨化炭黑固相萃取柱均购自博纳艾杰尔科技公司;Poly-sery固相萃取柱(德国CNW公司); 0.22 μm水相滤膜(上海安谱科学仪器有限公司)。

样品:清香型、浓香型、酱香型白酒,以及黄酒、干红葡萄酒均购自市场。

### 1.2 标准溶液的配制

分别称取10种有机酸标准品5 mg于5 mL容量瓶中,用超纯水进行溶解并定容,配制成质量浓度为1 mg/mL的单标准储备液,于-18 ℃下避光保存。用超纯水稀释并配制成为不同浓度的混合标准溶液,于4 ℃下冰箱冷藏保存。使用时用超纯水逐级稀释,配制成0.05、0.1、0.2、0.5、1.0、2.0、5.0、10.0 mg/L的系列混合标准溶液。

### 1.3 样品前处理方法

白酒:取白酒样品5 mL于15 mL离心管中,氮吹至1 mL后用4 mL超纯水复溶至5 mL,吸取复溶液1 mL稀释100倍,将复溶液及稀释后的溶液通过0.22 μm滤膜,收集滤液上机分析。

黄酒及干红葡萄酒样品:取黄酒及干红葡萄酒样品各10 mL于15 mL离心管中,在8000 r/min条件下离心10 min,将上清液移入新的离心管中待用。分别用10 mL甲醇、10 mL水活化石墨化炭黑固相萃取小柱,将黄酒及干红葡萄酒样品分别通过石墨化炭黑固相萃取小柱进行净化,弃去前3 mL流出液,收集后段流出液。将黄酒流出液用去离子水稀释50和200倍,将葡萄酒流出液用去离子水稀释100和400倍,通过0.22 μm滤膜,收集滤液上机分析。

### 1.4 检测条件

#### 1.4.1 色谱条件

色谱柱采用Dionex IonPac^TM^ AS11-HC型阴离子分析柱(250 mm×4 mm), Dionex IonPac^TM^ AS11-HC型保护柱(50 mm×4 mm),柱温30 ℃,检测池温度35 ℃,流速1.0 mL/min。流动相为由淋洗液自动发生器在线产生的KOH溶液。梯度洗脱程序为:0~15.0 min, 0.8 mmol/L KOH; 15.0~25.0 min, 0.8~50.0 mmol/L KOH; 25.0~30.0 min, 50.0~70.0 mmol/L KOH; 30.0~35.0 min, 70.0 mmol/L KOH; 35.0~35.1 min, 70.0~0.8 mmol/L; 35.1~38.0 min, 0.8 mmol/L KOH。进样量25 μL; Dionex ADRS 600 (4 mm)型阴离子抑制器,抑制电流175 mA。

#### 1.4.2 质谱条件

离子源:电喷雾电离(ESI)源;扫描方式:负离子模式扫描;多反应监测(MRM)模式;毛细管电压为3.10 kV;离子源温度为120 ℃;锥孔气流速为50 L/h;锥体电压50 V;雾化气体流量7.0 L/h;碰撞气体流量0.15 mL/min;碰撞池出口电位5 V;脱溶剂气温度为400 ℃,流速为850 L/h;采用MRM模式采集各标准样品的母离子及对应的响应较强的子离子,使用分析软件(MassLynx, 4.1版)进行系统控制和数据处理,10种有机酸的保留时间、母离子、子离子及锥孔电压(cone voltage)和碰撞能量(collision energy)等参数见表1。

**表1:** 10种有机酸的保留时间及质谱参数

No.	Compound	Retention time/min	Parent ion (m/z)	Product ion (m/z)	Cone voltage/V	Collision energy/eV
1	quinic acid	11.56	191.1	85.1^*^	38	21
				59.1		23
2	lactic acid	13.94	89.1	45.1^*^	22	11
				43.1		10
3	succinic acid	23.95	117.1	73.0^*^	19	12
				99.1		11
4	malic acid	23.97	133.1	115.0^*^	19	12
				71.0		16
5	tartaric acid	24.35	149.0	87.0^*^	20	12
				73.0		16
6	maleic acid	24.85	115.1	71.1^*^	15	15
				27.1		10
7	fumaric acid	25.94	115.1	71.0^*^	17	9
				27.0		11
8	oxalic acid	26.17	89.1	43.0^*^	20	12
				45.1		11
9	citric acid	28.36	191.0	87.0^*^	18	12
				111.0		20
10	aconitic acid	30.75	173.0	85.1^*^	12	7
				129.1		11

* Quantitative ion.

## 2 结果与讨论

### 2.1 前处理条件的优化

白酒属于蒸馏酒,杂质较少。但实验发现,若白酒稀释后直接进样,由于乙醇的存在会对乳酸的峰形造成干扰,使得乳酸的离子峰变宽,无法准确定量。本实验利用氮吹仪将白酒样品中的大量乙醇挥发去除^[19]^,再用去离子水进行稀释,进样检测。通过实验验证,10种有机酸回收率为88.3%~111.5%,回收率良好。

黄酒和干红葡萄酒相比于白酒组分较为复杂,黄酒中焦糖色色素为主要干扰物质;干红葡萄酒中色素、多酚类物质以及糖等都有可能对有机酸的检测产生干扰^[20]^。

为了提高有机酸的回收率,本实验首先对比了不同固相萃取柱对于干红葡萄酒的净化效果,选择了Poly-sery去除食品中着色剂专用SPE柱、IC-RP固相萃取柱和石墨化炭黑固相萃取小柱进行测定。发现采用Poly-sery柱以及IC-RP柱时,10种有机酸的回收率均较低,部分有机酸回收率低于70%(见图1),净化效果较差。石墨化炭黑柱对10种有机酸的回收率均良好,故选用石墨化炭黑固相萃取柱进行净化。随后,使用石墨化炭黑固相萃取柱对黄酒进行净化,结果表明,10种有机酸回收率为89.5%~107.2%,回收率较好。为实验方法统一,故选择石墨化炭黑固相萃取柱对黄酒及干红葡萄酒进行净化。

**图1:**
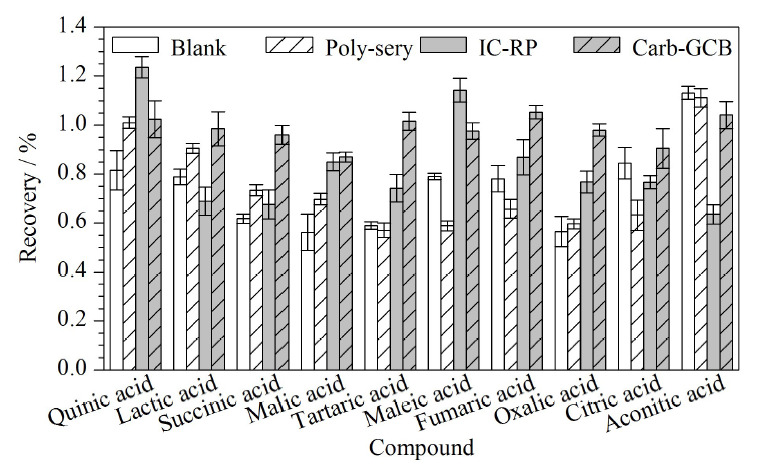
3种固相萃取柱对干红葡萄酒中10种有机酸回收率的影响(*n*=6)

### 2.2 离子色谱条件的优化

#### 2.2.1 色谱柱的选择

有机酸类物质属于极性物质,在反相C_18_色谱柱上保留较差,而Dionex IonPac^TM^AS11-HC型阴离子分析柱(250 mm×4 mm)和Dionex IonPac^TM^AS19型阴离子分析柱(250 mm×4 mm)都属于高柱容量色谱柱,具有较强的亲水性,对于有机酸这类极性物质具有良好的保留特性。AS11-HC色谱柱的固定相填料主要为高交联度的乙基乙烯基苯-二乙烯基苯共聚物(EVB-DVB)聚合物,AS19色谱柱的固定相填料主要为超孔型EVB-DVB颗粒,它们本身带有正电荷,对淋洗液中阴离子和样品阴离子的静电吸附作用不同。本文对比了AS11-HC和AS19色谱柱对于10种有机酸的分离情况,发现在使用AS11-HC色谱柱时,10种有机酸的分离效果更好,最终选择Dionex IonPac^TM^ AS11-HC型阴离子分析柱(250 mm×4 mm)进行分离。

#### 2.2.2 梯度洗脱程序的优化

有机酸类化合物具有酸性和亲水性,在较强的碱性溶液中以阴离子形式存在。离子色谱中的淋洗液发生器可以在线产生不同浓度的KOH溶液,消除人工配制淋洗液所带来的误差和可能的空气污染,保障了淋洗液的高纯度及稳定性,增强了系统的重现性和灵敏度^[21]^。因此,本文选择淋洗液发生器产生的KOH溶液作为淋洗液。

对于小分子有机酸,电荷数是影响其柱保留的主要因素。本实验中分离的有机酸包括一元、二元和三元羧酸,在水或稀碱液中会解离成一价、二价和三价阴离子,不同价态离子与离子交换剂亲和力不同,电荷越高,亲和力越大。因此,为了改善分离效果,达到最短时间内得到最佳分离的目的,本文考察不同浓度的KOH淋洗液对标准样品分析的影响,并进行梯度洗脱程序优化实验。奎尼酸和乳酸是一价无机阴离子,对离子交换剂的亲和力较弱,出峰时间较早,所以前15 min需要用低浓度(小于5 mmol/L)的KOH进行洗脱。故本实验前15 min选取了0.8、1.5、3.0 mmol/L的KOH对奎尼酸和乳酸的分离效果进行考察(见图2), 15 min后的淋洗液梯度统一为40~60 mmol/L KOH梯度洗脱。可以看到前15 min随着淋洗液浓度的增加,奎尼酸和乳酸的分离时间不断提前,分离度不断减小。结果表明,当初始淋洗液浓度为0.8 mmol/L KOH时两种酸的分离效果最好。

**图2:**
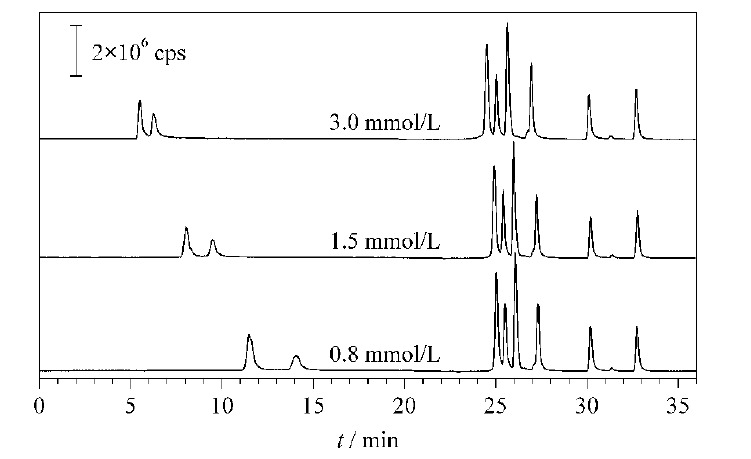
采用不同初始浓度淋洗液时10种有机酸的总离子流图

草酸、富马酸、马来酸、琥珀酸、苹果酸、酒石酸和乌头酸是二价无机阴离子,柠檬酸为三价无机阴离子,对离子交换剂的亲和力较强,出峰时间较晚,所以20 min后需要用较高浓度的KOH进行洗脱。故考察15 min后不同梯度程序对8种酸的分离影响(见图3)。结果表明,随着淋洗液浓度的增加,8种有机酸的出峰时间均提前,分离效果相近。当选用图3a的梯度程序时,10种有机酸的整体出峰时间为38 min,而选用图3c的梯度程序时,整体出峰时间为32 min,为了实现快速分离检测,故选择图3c的梯度程序进行洗脱分离。

**图3:**
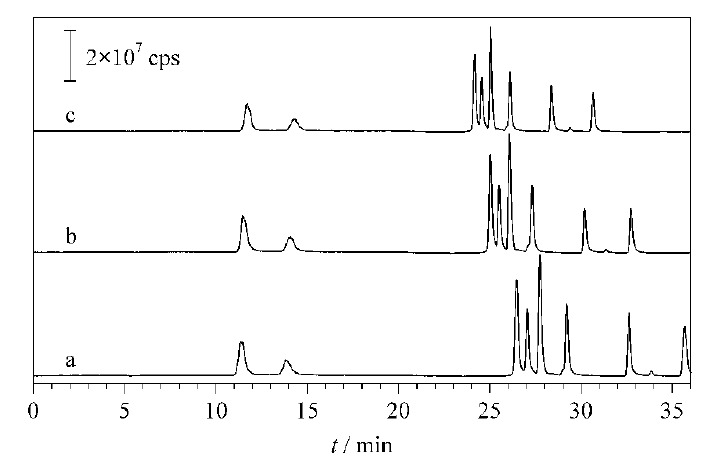
采用不同淋洗液梯度时10种有机酸的总离子流图

#### 2.2.3 流速的选择

在确定了分析柱和淋洗液的情况下,对流速进行了优化。分别考察了流速0.8、1.0、1.2 mL/min对分离效果的影响。实验结果表明,流速越高,分析时间越短,但分离度会受到一定影响。当流速为1.2 mL/min时,出峰时间较为靠前,但柱压接近2.0×10^7^ Pa上限,长时间高压检测,对色谱柱及仪器的使用寿命有较大影响^[22]^。当流速为0.8 mL/min时,发现色谱峰峰形较宽。综合测定时间、响应值、色谱柱使用寿命以及峰形,最终确定流速为1.0 mL/min。

### 2.3 离子色谱与质谱连接方式的优化

离子色谱淋洗液中含有KOH,当其进入到ESI源时无法完全挥发,进而堆积在电喷雾喷嘴处,对仪器部件产生腐蚀,对仪器的损伤较大。在测定时需要避免难挥发缓冲盐进入质谱检测器。离子色谱中的电解抑制器可以实现在线除盐,消除淋洗液中的盐或碱对ESI源的影响和对质谱部件的损害。通过选择ADRS 600 (4 mm)型抑制器,调节抑制器的电流,使抑制器电解水产生H^+^,通过阳离子交换膜使K^+^与H^+^进行交换然后排至废液。最终进入质谱的为经过抑制器抑制后的产物纯水^[23]^。

本实验选择使用AS11-HC型号色谱柱进行分离待测物,最佳流速为1.0 mL/min,而质谱的最适流速为0.3~0.5 mL/min,若以1.0 mL/min的流速直接进入质谱检测器会使溶剂挥发不完全,而未雾化的液滴还有可能进入质谱,污染质谱内部,使背景噪声增大,影响检测。所以本实验在进入质谱检测器前安装了一个三通进行分流,使进入质谱的流速保持在0.5 mL/min,将分流出的一部分淋洗液回流至抑制器中进行再生。在保证被测物质良好分离的情况下,降低背景噪声,同时以适于质谱检测的流速进入质谱,得到良好的检测结果。仪器连接方式见图4。

**图4:**
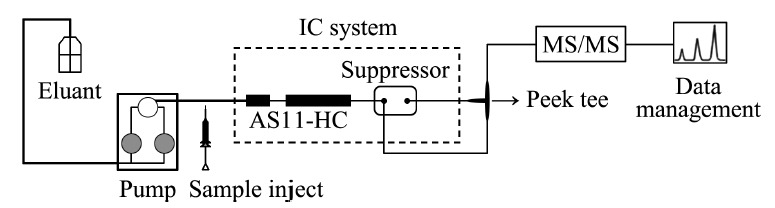
仪器连接示意图

### 2.4 质谱条件的选择与优化

本实验选择三重四极杆质谱作为检测器测定样品的含量,其抗干扰能力强,灵敏度高,能准确进行定性和定量。采用注射泵以流动注射的方式分别在正离子和负离子模式下对1 mg/L的10种有机酸标准溶液进行全扫描,结果表明:有机酸标准样品在负离子模式下响应值较好。锥孔电压和碰撞能量是影响质谱测定结果的重要因素,锥孔电压直接影响各组分的灵敏度,过高的锥孔电压会导致分子离子在离子源内发生碰撞解离,从而影响母离子的丰度,进而影响检出限和灵敏度。通过全扫描逐个进行前体离子扫描,根据离子信号强度选出目标前体离子,母离子为[M-H]^-^型,利用仪器手动优化功能,分别对锥孔电压和碰撞能量等进行优化,同时考察雾化气温度,毛细管温度等指标,确定测定的最佳质谱条件。10种有机酸的MRM色谱图见图5。

**图5:**
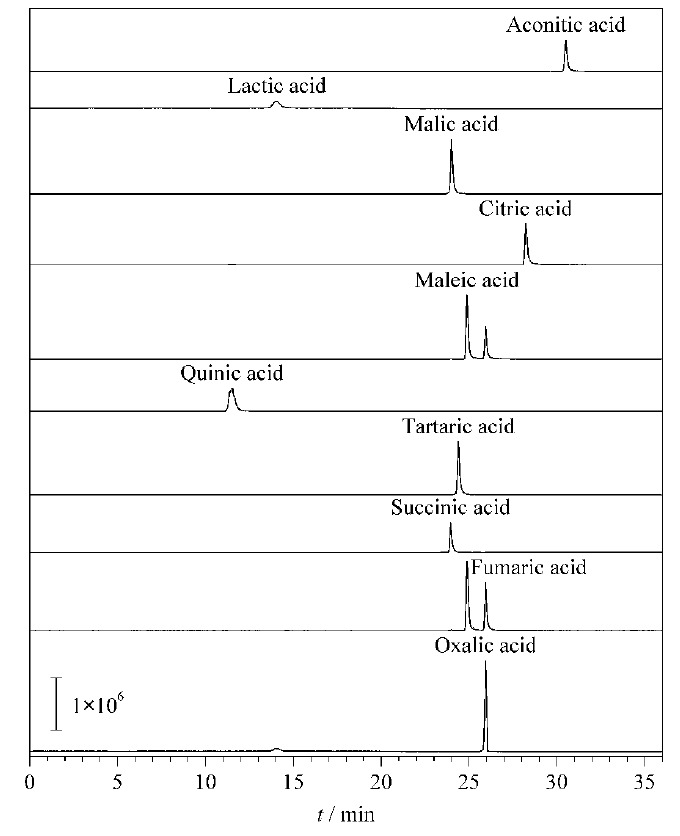
10种有机酸的MRM色谱图

### 2.5 方法学评价

#### 2.5.1 线性范围和检出限

将10种有机酸的标准储备液配制成不同浓度的混合标准工作液。按前文1.4.1和1.4.2节中所述的离子色谱和质谱条件进行测定。以10种有机酸的质量浓度为横坐标,色谱峰面积为纵坐标,绘制标准工作曲线,见表2。可以发现,10种有机酸在各自的质量浓度范围内线性关系良好,相关系数(*r*^2^)均大于0.99。以色谱峰信噪比*S/N*=3和*S/N*=10的浓度分别确定方法的检出限(LOD)以及定量限(LOQ), 10种有机酸的检出限在1.0~8.0 μg/L之间,定量限在3.5~26.5 μg/L之间。

**表2:** 10种有机酸的线性范围、线性方程、相关系数、检出限和定量限

No.	Compound	Linear range/(mg/L)	Linear equation	r^2^	LOD/(μg/L)	LOQ/(μg/L)
1	quinic acid	0.05-2	Y=3.71×10^5^X+1.97×10^4^	0.9939	4.0	12.5
2	lactic acid	0.05-10	Y=3.30×10^4^X+3.08×10^3^	0.9955	8.0	26.5
3	succinic acid	0.05-5	Y=2.70×10^5^X+5.52×10^4^	0.9957	1.5	5.0
4	malic acid	0.05-2	Y=4.49×10^5^X+3.16×10^4^	0.9925	1.5	5.0
5	tartaric acid	0.05-2	Y=2.56×10^5^X+1.22×10^4^	0.9964	1.0	3.5
6	maleic acid	0.05-2	Y=6.56×10^5^X+1.00×10^4^	0.9960	1.5	5.0
7	fumaric acid	0.05-2	Y=2.75×10^5^X+1.72×10^4^	0.9930	3.5	12.0
8	oxalic acid	0.05-2	Y=2.28×10^4^X+3.78×10^3^	0.9942	3.5	12.0
9	citric acid	0.05-2	Y=2.53×10^5^X+9.34×10^3^	0.9988	3.0	10.0
10	aconitic acid	0.05-2	Y=4.15×10^5^X+1.29×10^4^	0.9951	4.0	13.5

*Y*: peak area; *X*: mass concentration, mg/L.

#### 2.5.2 精密度和回收率

将低、中、高3个水平的适量混合标准溶液加入到3种酒类样品中,进行加标回收测定,每个加标水平测定6次,回收率和精密度结果如表3所示。白酒、黄酒和干红葡萄酒样品中10种有机酸在低、中、高3个加标水平下均有较好的准确度和精密度。平均加标回收率为83.0%~112.1%,相对标准偏差≤ 9.1%,可用于3种酒样品中10种有机酸含量的测定。

**表3:** 10种有机酸在不同添加水平下的回收率和相对标准偏差(*n*=6)

Compound	Liquor			Yellow rice wine			Dry red wine		
	Spiked/(mg/L)	Recovery/%	RSD/%	Spiked/(mg/L)	Recovery/%	RSD/%	Spiked/(mg/L)	Recovery/%	RSD/%
Quinic acid	0.1	96.0	5.6	0.1	95.7	5.8	0.1	108.8	5.8
	0.2	102.0	2.3	0.2	99.2	1.9	0.2	95.5	6.4
	0.5	105.4	3.3	0.5	101.4	2.7	0.5	102.4	3.1
Lactic acid	1	110.2	3.7	1.0	99.8	5.9	1.0	103.9	1.8
	2	98.9	4.1	2.0	105.2	1.6	2.0	102.4	2.3
	4	99.3	2.9	4.0	101.8	4.9	4.0	105.8	5.6
Succinic acid	0.1	103.1	3.5	0.1	104.6	1.3	0.1	98.7	2.6
	0.2	107.0	4.3	0.2	89.5	2.3	0.2	96.5	0.5
	0.5	108.4	5.4	0.5	96.4	6.7	0.5	95.0	2.3
Malic acid	0.1	112.0	4.8	0.1	100.5	4.4	0.1	88.2	5.4
	0.2	105.0	3.7	0.2	97.8	4.9	0.2	87.0	6.2
	0.5	107.8	4.2	0.5	103.7	5.8	0.5	106.2	2.8
Tartaric acid	0.1	94.0	6.3	0.1	102.3	7.2	0.1	93.2	1.8
	0.2	101.0	6.0	0.2	97.8	5.5	0.2	84.1	8.9
	0.5	96.2	5.8	0.5	101.1	4.9	0.5	101.6	3.7
Maleic acid	0.1	84.4	3.1	0.1	104.2	1.3	0.1	98.3	3.7
	0.2	105.3	6.8	0.2	99.7	6.3	0.2	102.4	4.1
	0.5	98.8	5.1	0.5	107.2	4.7	0.5	89.2	4.9
Fumaric acid	0.1	105.0	3.4	0.1	99.0	3.3	0.1	105.3	1.6
	0.2	111.5	2.4	0.2	107.2	3.7	0.2	93.0	2.1
	0.5	100.6	2.6	0.5	101.3	8.5	0.5	111.6	4.1
Oxalic acid	0.1	101.1	3.7	0.1	101.0	4.7	0.1	107.0	2.1
	0.2	96.5	5.6	0.2	103.5	6.2	0.2	107.5	3.6
	0.5	102.4	8.0	0.5	98.4	5.8	0.5	98.0	2.1
Citric acid	0.1	88.2	7.4	0.1	90.3	3.9	0.1	106.8	5.1
	0.2	97.5	4.7	0.2	93.6	4.2	0.2	90.5	4.5
	0.5	101.2	6.2	0.5	100.7	3.8	0.5	83.0	6.8
Aconitic acid	0.1	94.2	5.8	0.1	103.0	4.3	0.1	112.1	9.0
	0.2	108.5	3.6	0.2	90.5	5.6	0.2	91.2	9.1
	0.5	91.2	5.9	0.5	92.8	4.4	0.5	104.1	7.2

### 2.6 实际样品的检测

采用所建立的方法对市场上购买的白酒(清香型、浓香型和酱香型)、黄酒、干红葡萄酒进行测定(见表4),经过不同稀释倍数换算后,得到3种酒类样品中10种有机酸的测定值。结果表明,白酒中,酱香型白酒含有的有机酸种类较多,所以酱香型白酒相对于其他两种香型白酒口感更为丰富^[24]^, 3种香型的白酒中,乳酸含量均为最高,其中酱香型白酒中乳酸含量最高。黄酒与干红葡萄酒中均含有8种有机酸,其中黄酒中乳酸及琥珀酸含量较高,干红葡萄酒中乳酸、琥珀酸及酒石酸含量较高。

**表4:** 酒样品中10种有机酸的含量(n=3)

Compound	Mild-flavor Chinese spirits		Strong-flavor Chinese spirits		Jiang-flavor Chinese spirits		Yellow rice wine		Dry red wine		
Quinic acid	ND		ND		ND		28.35±	0.65	13.43±		0.90
Lactic acid	253.82±	1.76	563.13±	10.55	918.34±	5.44	2092.4±	39.85	1569.13±		48.49
Succinic acid	0.74±	0.08	0.66±	0.04	2.58±	0.13	978.42±	12.92	375.13±		2.50
Malic acid	ND		0.05±	0.01	0.29±	0.02	36.85±	0.94	248.26±		8.43
Tartaric acid	ND		0.06±	0.01	0.09±	0.01	43.93±	0.78	822.24±		11.34
Maleic acid	0.06±	0.01	0.44±	0.04	1.70±	0.03	44.33±	1.57	7.90±		0.13
Fumaric acid	ND		ND		0.45±	0.03	ND		N		D
Oxalic acid	ND		ND		ND		4.55±	0.13	31.80±		3.67
Citric acid	0.06±	0.01	0.03±	0.01	0.05±	0.01	41.23±	1.49	193.10±		4.24
Aconitic acid	ND		ND		ND		ND		N		D

Values are mean±SD. ND: not found.

## 3 结论

本文搭建了离子色谱-三重四极杆质谱检测平台,并建立了一种固相萃取结合离子色谱-串联质谱同时测定白酒、黄酒、干红葡萄酒3种酒类样品中10种有机酸的检测方法。采用阴离子分析柱解决了常规方法中极性物质不易保留的难题;采用质谱对有机酸进行定性与定量,与常规方法相比,提高了抗干扰能力与灵敏度,同时避免了假阳性结果的出现。对实际样品的检测表明,该方法能够满足3种酒样品中10种有机酸的分析要求,为不同酒品中有机酸的定性和定量分析提供了基础数据与技术支持。
